# In-hospital cardiac arrest due to sepsis – Aetiologies and outcomes in a Swedish cohort study

**DOI:** 10.1016/j.resplu.2023.100492

**Published:** 2023-11-01

**Authors:** Samuel Bruchfeld, Ingrid Ronnow, Felix Bergvich, Frida Brochs, Matilda Fahlen, Kristoffer Strålin, Therese Djärv

**Affiliations:** a Department of Medicine Solna, Karolinska Institutet, Stockholm, Sweden; b Emergency Department, Karolinska University Hospital, Stockholm, Sweden; c Department of Medicine Huddinge, Karolinska Institutet, Stockholm, Sweden; d Department of Infectious Diseases, Karolinska University Hospital, Stockholm, Sweden

**Keywords:** IHCA, Aetiologies, Septic shock

## Abstract

**Objectives:**

Awareness of causes of cardiac arrest is essential to prevent them. A recent review found that almost every sixth in-hospital cardiac arrest is caused by infection. Few studies have explored how infections cause cardiac arrest.

**Aim:**

To describe the features, mechanisms and outcome of sepsis-related cardiac arrests.

**Material and methods:**

All patients ≥18 years who suffered a cardiac arrest at Karolinska University Hospital between 2007 and 2022 with sepsis as the primary cause were included. Data were collected the Swedish Registry for Cardiopulmonary Resuscitation and medical records. The primary outcome was survival to discharge.

**Results:**

Out of 2,327 in-hospital cardiac arrests, 5% (*n* = 123) suffered it due to sepsis, and 17% (21) survived to hospital discharge. Two thirds of patients were admitted to the hospital due to sepsis and suffered their cardiac arrest after a median of four days. About half (*n* = 59) had deranged vital signs before the event. Most were witnessed in general wards. In all, 47% (*n* = 58) had asystole and 24% (*n* = 30) as the first heart rhythm. The respiratory tract was the most common source of infection. Most patients were undergoing antibiotic therapy and one third had a positive microbiological culture with mixed gram-positive bacteria or Escherichia coli in the urine.

**Conclusion:**

Our results suggest that sepsis is an uncommon and not increasing cause of in-hospital cardiac arrest and its outcome is in line with other non-shockable cardiac arrests. Deranged respiratory and/or circulatory vital signs precede the event.

## Introduction

Resuscitation of in-hospital cardiac arrest (IHCA) is estimated to occur in 290,000 hospitalised patients annually in the US, and about 2,700 IHCAs are reported yearly in Sweden, with an overall 30-day survival of 25%.^1^, ^2^, ^3^

Preventing IHCA is a key step in the chain of survival, but in order to prevent IHCA, the causes and mechanisms must be understood. A recent systematic review found that 14% of all IHCAs are caused by infection.^4^ Included studies report that infection and sepsis are potential clinical conditions contributing to hypoxic or hypovolemic cardiac arrest.^5^, ^6^ Sepsis is a life-threatening organ dysfunction caused by a dysregulated host response to infection, while septic shock has a particularly profound circulatory abnormality.^7^ Therefore, abnormal vital signs, namely high respiratory rate and low blood pressure, are key features of bedside identification of sepsis as well as prognostic for outcome.^7^ Even though sepsis is often identified by bedside criteria, blood culture sampling is a cornerstone of sepsis management, and blood culture is widely accepted as the gold standard for microbiological diagnostics in sepsis.^8^ Understanding and targeting sepsis-**related** pathophysiology before, during and after cardiac arrest may therefore have the potential to improve outcomes.^9^ Yet, very few studies have explored the mechanism for how and in whom an infection/sepsis causes IHCA. Therefore, our aim was to shed light on the mechanisms of and outcomes in IHCA caused by sepsis.

## Method

### Study design

This hospital-based cohort study used the Swedish Registry for Cardio-Pulmonary Resuscitation (SRCR) as the main source to identify all IHCAs at Karolinska University Hospital.

### Settings

Karolinska University Hospital is one of five large hospitals in Stockholm (home to approximately two million people) and has two equally sized sites, 30 kilometres apart: Solna and Huddinge. The Solna site is a level one trauma unit and has neuro- and thoracic surgery units. The Huddinge site includes a geriatric ward and relatively fewer intensive care unit (ICU) beds. Our hospital has four levels of care, ranging from ICU, High Dependency Units, Higher level of care units to general wards. Since October 2017, a sepsis alert is activated at the emergency department triage when signs of organ dysfunction are combined with signs of infection (i.e. fever, history of fever, or clinical suspicion of infection).^8^ Patients who trigger the sepsis alert are subjected to a multidisciplinary bedside assessment by physicians from the Emergency Department, the Department of Infectious Diseases and the Intensive Care Unit within 15 minutes to optimize clinical assessment and treatment. Karolinska has about 1,300 beds and approximately 108,000 admissions and 1.8 million patient visits each year.

### Ethics

All patients surviving IHCA were asked for informed consent six months afterwards and agreed to participate in the SRCR. The Regional Ethical Review Board in Stockholm, Sweden approved the study (ref. 2022-02967-01).

### Study population

We defined IHCA according to the SRCR^10^, ^11^ as “a hospitalised patient who is unresponsive with apnoea (or agonal, gasping respiration) where CPR and/or defibrillation have been initiated.” All adult (18 years) patients who had an IHCA between 1 January 2007 and 31 December 2022 at Karolinska University Hospital were included. No adult patients nor specific locations for the IHCA were excluded. In the case of multiple IHCAs, only the first event per year was included. Patients with a do-not-resuscitate order or where the team terminated CPR early because of a poor prognosis were excluded.

### Definitions and data collection

#### Cardiac arrest and patient factors

Patients were identified through cases the hospital reported to the SRCR, where data on the following variables were collected: sex, age (in years), location of IHCA (patient ward, intermediate care unit, intensive care unit (ICU), angiography lab/operating theatre or other areas, including emergency department and radiology department) and first documented heart rhythm (shockable, i.e. VT/VF, or non-shockable, i.e. PEA/asystole). Thereafter, information on comorbidities was identified by entering the hospital’s electronic patient record (Take Care version 14.2.9) (ICD-10 codes available at admission to the hospital and assessed according to the Charlson Comorbidity Index (CCI) by ICD codes as described by Ludvigsson et al.).^12^, ^13^, ^14^, ^15^ Vital signs were drawn from the medical file up to 12 hours prior to the IHCA; 12 hours was selected due to the longest range between controls recommended in the National Early Warning Score (NEWS).^12^

#### Infection as the cause of IHCA

**Fig. 1:**
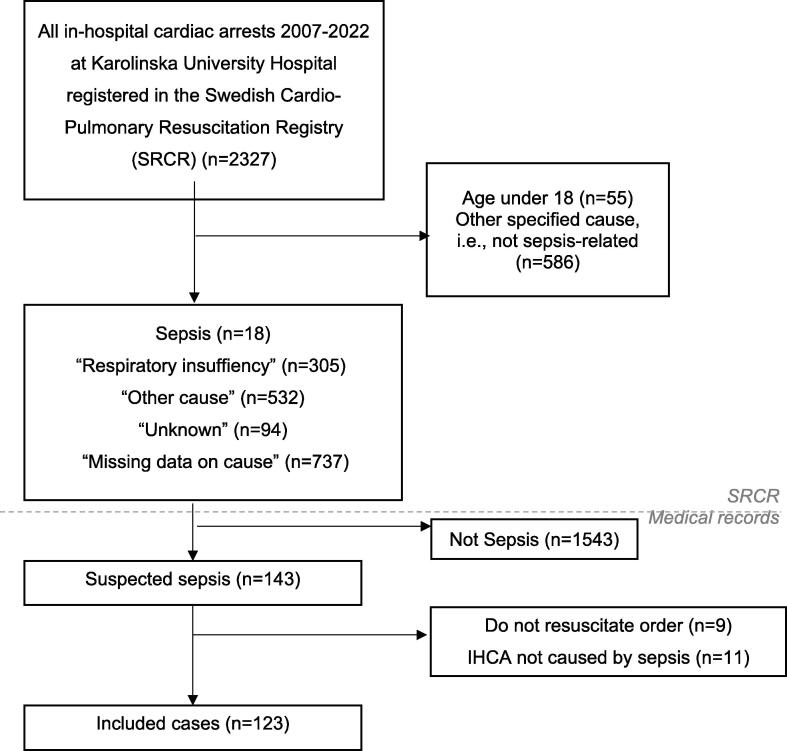
Flow Chart of Patient Inclusion, In-hospital cardiac arrest at Karolinska University Hospital 2007–2022.

Within the SRCR, aetiology is specified either as a prespecified category or as “other” with the opportunity to provide a free text answer. The following prespecified categories in the SRCR were included: “sepsis”, “other respiratory failure”, “unknown” and “other”. Manual screening of medical records was performed for all included cases in order to exclude cases of IHCA that had a non-infectious aetiology and only include patients with sepsis in the final cohort (Fig. 1). The definition of sepsis as a cause of IHCA was defined as any free text comment by the treating physician in the medical record stating suspected sepsis, sepsis, septic shock or ICD code sepsis 14 days prior to the IHCA*.*

#### Microbiological cultures

Culture results from samples taken 14 days prior to the IHCA from blood, urine, sputum, NPH, joint fluid, faeces and central vein catheter (CVC) were screened for eligibility. In cases where multiple cultures were taken from the same site but at different dates, only the last cultures prior to the IHCA were recorded. All types of pathogens were included in the study. Microbiological culture results were classified in five groups regardless of culture site: gram-positive culture results, gram-negative culture results, mixed culture results, negative culture results and missing culture results. Mixed culture results were defined as a combination of either gram-positive, gram-negative, fungal culture or virus culture. Culture-negative sepsis was defined as sepsis without positive evidence of pathogens from any culture.

#### Outcome

The primary outcome was survival to hospital discharge as recorded in the SRCR. The electronic patient record is automatically linked to the Swedish Total Population Register, which enables a national, all-encompassing follow-up regarding survival.^13^ Secondary outcomes were: alive at the end of CPR (yes/no), and change in CPC score between admission and discharge.

### Statistical analysis

Continuous and discrete variables were reported as medians with interquartile or total range. Patients with missing data were excluded from analysis, including the missing variables, and no data was imputed or estimated. All statistical analyses were done using STATA (version 13).

## Results

Out of a total of 2,327 patients who suffered an IHCA at Karolinska between 2007–2022, 1,686 (72%) were selected for chart review. After reviewing medical records, 123 (5% of 2,327) patients fulfilled our definition of sepsis as the cause of an IHCA (Fig. 1).

### Patient characteristics

**Fig. 2:**
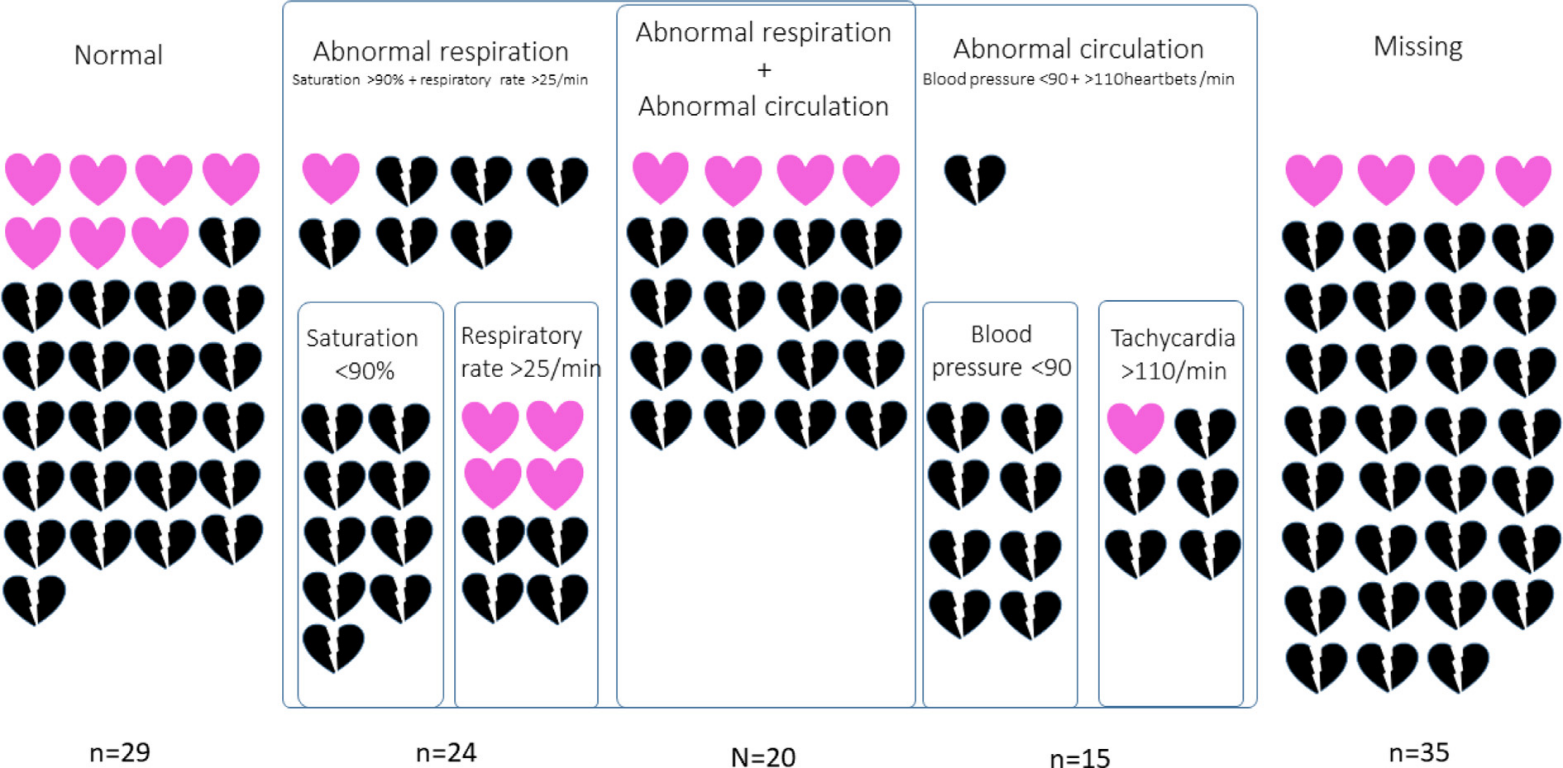
Presence of abnormal respiratory and/or circulatory vital signs up to 12 hours before sepsis-related in-hospital cardiac arrest among 123 patients at Karolinska University Hospital 2007–2022. *Pink heart- 30 day survivor, black broken heart-dead before 30 days.

Of the 123 patients included in the final analysis, 85 (69%) were male and the median age was 70 years (IQR 63–77), (Table 1). A total of 109 (88%) had at least a moderately high score on the Charlson Comorbidity Index. Two thirds were admitted to hospital due to sepsis or an infection. The median time from admission to IHCA was four days (IQR 1–10). However, one third suffered an IHCA within the first 24 hours after admission. The median SOFA-score was 4 (IQR 3–6) and most patients scored between 3–5. Regarding the presence of deranged vital signs before the IHCA, both respiratory and circulatory deranged vital signs, as well as a combination of the two, were found even though missing vital signs was the most common finding (Fig. 2). Among 88 patients with vital signs registered within 12 h prior to the IHCA, 30-day survival was 7/29 (24%) among those with normal vital signs and 10/59 (17%) among those with abnormal vital signs (*p* = 0.42, Fig. 2).

**Table 1:** Characteristics and intra-arrest factors in patients having an sepsis-related in-hospital cardiac arrest at Karolinska University Hospital 2007–2022.

**Characteristic**	**30-day survival** ***(n = 21)***	**No 30-day survival** ***(n = 102)***
**Age, median (IQR)**	69 (61–75)	71 (64–78)
**Male**	14 (67)	71 (70)
**Charlson Comorbidity Index**		
None (0 points)	2 (10)	2 (2)
Low 1–2 points	4 (19)	6 (6)
Moderate 3–5 points	10 (48)	43 (42)
High at least 6 points	5 (24)	51 (50)
**Days from admission to cardiac arrest, median (IQR)**	4(0–8)	4 (1–13)
**Admission cause**		
Planned admission	3 (14)	3 (14)
Infectious cause	15 (71)	64 (63)
Medical cause	2 (10)	22 (22)
Surgical cause	1 (5)	11 (2)
Other cause	-	2 (2)
**Sequential organ failure assessment (SOFA)**		
0points	–	3 (3)
1–2 points	6 (29)	20 (20)
3–5 points	14 (67)	46 (45)
At least 6 points	1 (5)	30 (29)
Missing	–	3 (3)
**National Early Warning Score up to 12 hours before the IHCA**		
0–4 points “Low risk”	6 (29)	20 (20)
3p in one parameter “Low-medium risk”	2 (10)	6 (6)
5-6points “Medium risk”	5 (24)	17 (17)
At least 7 points “High risk”	4 (19)	28 (27)
Missing	4 (19)	31 (30)
**Location of the IHCA**		
ICU/IMCU/CCU	6 (29)	24 (24)
Emergency room	2 (10)	8 (8)
Ordinary ward	11 (50)	68 (67)
Other incl Catheterisation lab/operating room	2 (10)	3 (14)
**ECG monitoring**	10 (48)	50 (49)
**Witnessed**	20 (95)	71 (70)
**Initial rhythm**		
PEA	7 (33)	23 (23)
Asystole	5 (24)	53 (52)
VF/VT	2 (10)	7 (7)
**ROSC**	21 (100)	32 (31)
Time to ROSC, median (IQR)**^f^**	7 (4–10)	10 (5–20)
Time to termination of resuscitation without ROSC, median (IQR)	NA	25 (17–35)

Abbreviations: CCU – cardiac critical care unit, ICU – intensive care unit, IMCU – intermediate care unit, IQR – interquartile range, PEA – pulseless electrical activity, ROSC – return of spontaneous circulation, VF – ventricular fibrillation, VT – ventricular tachycardia.

### Intra-arrest factors

Most patients suffered cardiac arrest in wards (*n* = 79 64%): half were ECG monitored and the vast majority were witnessed (Table 1). The initial rhythm was asystole in half of the patients and a quarter had PEA, whereas only 9 (7%) had a first shockable rhythm. Duration of CPR was in median 20 minutes (IQR 8–25). In all, 80% (99) was given adrenaline, 55% (6 out of 11 with initial shockable rhythm) was given amiodarone intra-arrest. Those that were not administered any drugs had CPR durations less than 6 minutes.

### Factors related to sepsis

The most frequent site of infection was the lower respiratory tract (*n* = 44, 36%). The second most common site of infection (25% of patients) was unknown/undefined (Table 2). Almost all patients received antibiotics prior to the IHCA. In all, 115 (93%) had a microbiological culture prior to the IHCA, and 42 (37% out of the 115) revealed gram-positive bacteria (Table 2). Gram-positive bacteria were found in one third of the blood cultures and one fifth of the NPH cultures, while a quarter of the urine cultures contained gram-negative bacteria, mainly Escherichia coli (Supplementary Table A).

**Table 2:** Characteristics of the infections in patients having an infection-related in-hospital cardiac arrest at Karolinska University Hospital 2007–2022.

**Variable**	**30 day survival** **(*n* = 21)**	**No 30 day survival(*n* = 102)**
**Infection in organ system**		
Respiratory tract	9 (43)	35 (34)
Genitourinary tract	3 (14)	12 (12)
Skin	2 (10)	14 (14)
Intra-abdominal	2 (10)	11 (11)
Catheter related bloodstream	–	2 (2)
Undefined	5 (24)	28 (28)
**Given antibiotics before CA**	18 (86)	93 (91)
**Type of microorganism**		
G + bacteria	8 (38)	34 (33)
G- bacteria	3 (14)	24 (24)
Mixed	6 (29)	24 (24)
Negative	2 (10)	14 (14)
**Blood cultures**		
Positive culture	8 (38)	43 (42)
Negative culture	10 (48)	46 (45)
Three most common microbes		
-Escherichia coli G-	2 (10)	8 (8)
-Streptococcus pneumonia G+	1 (5)	6 (6)
-Staphylococcus aureus G+	1 (5)	6 (6)
**Urine cultures**		
Positive	5 (24)	34 (33)
Negative	10 (48)	38 (37)
Three most common microbes		
-Escherichia coli -	12 (57)	1 (<1)
-Klebsiella pneumonia	2 (10)	3 (3)
-Antigen of pneumococcal +	–	3 (3)
**Nasopharyngeal cultures**		
Positive	2 (10)	9 (9)
Negative	3 (14)	26 (25)
Two most common microbes		
-Staphylococcus aureus G+	2 (10)	2 (2)
-Streptococcus pneumonia G+	– (–)	2 (2)

^a^Right ventricular dilatation in all cases, and right heart mobile thrombus in one case. **^b^** % of patients with recorded ECG. Abbreviations: CTPA – computed tomography pulmonary angiogram, PE – pulmonary embolism.

### Survival and neurological function

In all, 53 patients had ROSC and 21 (17% out of 123) patients survived to 30 days. No clear visual trend in survival or percentage of cases was seen (Supplementary Fig. A). The sample size only allows for descriptive indications for the microbe type; for example, Escherichia coli was the single most common pathogen; 10 patients were infected with Escherichia coli in the blood and 8 patients of these patients did not survive to 30 days; another 13 patients were positive in the urine, 12 of which did not survive to 30 days (Supplementary Table A).

Neurological function at discharge was the same as at admission in 14 (66% of survivors and 82% of those with information) patients (Supplementary Table B). In all, two patients had a lower CPC score at discharge than admission; one patient had a CPC of one at discharge but had a missing CPC at admission.

## Discussion

This cohort study is the first to describe features of IHCA caused by sepsis, which was found in 5% of patients. Survival for these patients was 17%. Vital signs were often missing or showed affected respiration and or circulation prior to the IHCA. The high rate of missing vital signs may partly be explained by the fact that the National Early Warning Score was implemented in 2016 at Karolinska University Hospital, while this cohort includes cases from as far back as 2007. This is noteworthy as most sepsis-related IHCAs occurred in ordinary wards and not higher levels of care, and it is possible that transferring the patient to a higher level of care may have prevented the IHCA.

The prevalence found in the present study is slightly less than previous reports (7%–22%),^4^ which may relate to our strict inclusion criteria. For example, patients who would be considered to have sepsis based on SOFA scores or clinical status and microbiological cultures^8^ were not included in this study unless the clinicians who treated the patients stated such in the medical file. Further, we only included patients with sepsis, meaning that not all patients with infection were included since we were interested in the cause of IHCA rather than co-existing conditions. It is worth noting that numerous studies report the proportions and outcomes of patients with sepsis who experience an IHCA^14^ for any reason, such as a type 2 myocardial infarction by hypoxia, but not vice versa, i.e. the proportion of IHCA due to sepsis. These studies do not clearly define the entity but include pneumonia, septicaemia, sepsis syndrome with unspecified criteria and other infectious disease as a primary diagnosis leading to IHCA.^9^ Further, it is possible that previous studies overestimate the incidence of sepsis by using ICD-9 codes at discharge from the hospital. In summary, we believe that our strict criteria might have missed some septic patients, especially those who never had ROSC but most likely, since we manually screened all medical files for the 737 patients with missing/unknown cause in the registry, we believe that those patients having ROSC and/or those who survived to discharge should have been identified by clinicians.

Even though awareness of sepsis has increased, we could not discern a clear trend over time in either the percentage of IHCA caused by sepsis or in survival. Our survival ratio was similar to previous studies and is comparable to the overall survival for non-shockable IHCA in Sweden,^2^, ^3^ as well as older reports on IHCA survival capturing sepsis.^15^, ^16^

Respiratory tract infection was the most common source of infection, which is consistent both with the most common source of infection in the sepsis population at Karolinska University Hospital^8^ and with a previous study on IHCA in cohorts of septic patients.^14^ In this context, it is worth noting that we manually screened for sepsis among those reported to have respiratory insufficiency as the cause of IHCA in SRCR. Of note, all cases with hypoxia, or desaturation, prior to IHCA died in our cohort.

When examining circulatory mechanisms as the cause of sepsis-related IHCA, even though our sample is small, all patients with low blood pressure prior to IHCA died. Other studies have speculated that the reason for this relates to the fact that CPR is less effective.^9^ Tachycardia prior to IHCA was quite rare in our cohort. It is possible that this relates to myocardial depression and septic dysfunction,^9^ but this was not investigated in the present study.

Given the fact that arrhythmias, such tachycardia, were uncommon prior to the IHCA and the low number of shockable first rhythms, it can be assumed that the mechanisms behind sepsis-related IHCA are hypoxaemia and/or hypotension.

It is interesting that one third of the arrest occurred within 24 hours, this might relate to difficulties in assessing both severity, changes in clinical conditions as well as when escalation of care is needed. Our group has recently published data on rapid response team and their actions before an IHCA,^17^ and even if they are highly skilled in assessment of critically ill patients signs of deranged respiration might still have been underestimated regarding the risk of having an IHCA. Early after admission, it is possible that there is a false believe that giving patients initial adequate treatment might improve status and that could be waited for. Another recent study by our group has explored IHCA occurring in the ICU,^18^ even if the main focus was not causes of ICU-CA that study found that most were postoperative and re-arrest in prehospital or in-hospital cardiac arrests.

Half of the patients in the present study had positive blood culture results, which is higher than in the total sepsis cohort,^8^ but our finding that the three most common pathogens isolated from blood cultures were Escherichia coli, streptococcus pneumoniae and staphylococcus aureus is similar to findings in the sepsis cohort in our hospital^8^ and other previous studies.^19^ In this context it is important to note that staphylococcus aureus is an opportunistic pathogen and asymptomatic colonizer of human anterior nares and approximately 30% of individuals are permanently colonized,^20^ but it is an opportunistic pathogen^21^ and one of the leading pathogens causing bloodstream infection and sepsis. Patients at particular risk for Staphylococcus aureus sepsis are older and more comorbid,^21^ which could explain the common finding in our cohort.

Major limitations in the present study include the retrospective design and small sample size, which is limited by a lower incidence compared to previous studies. The small number limits subgroup descriptions and analysis such as different locations. A clinical criterion to characterize a septic patient, such as an SOFA score, is recommended.^7^ Unfortunately, SOFA scores were only accurately registered in four patients in our study, which likely relates to the gradual implementation from 2010 and onwards in Swedish medical records and the implementation of the National Early Warning Score in 2016 at Karolinska University Hospital.

Our study shed light on the mechanisms of sepsis-related IHCA by identifying a broad range of sources and microbes causing IHCA via both respiratory and circulatory mechanisms. Future studies may be needed to compare a cohort with an IHCA to the remaining admitted patients with sepsis but without an IHCA in order to identify risk patients and risk factors.

Finally, we would like to highlight that screening systems like NEWS, especially if the escalation plan is followed, might be a way forward to prevent IHCA.

In conclusion, our results suggest that sepsis is an uncommon, not increasing cause of IHCA and patient outcomes are in line with other non-shockable IHCA. Deranged respiratory and/ or circulatory vital signs precede the IHCA.

## Data sharing statement

No additional data exists that is suitable for publication since data are based on the medical records of individuals.

## CRediT authorship contribution statement

**Samuel Bruchfeld:** Conceptualization, Methodology, Investigation, Visualization. **Ingrid Ronnow:** Conceptualization, Methodology, Formal analysis, Writing – review & editing, Visualization. **Felix Bergvich:** Conceptualization, Methodology, Writing – review & editing. **Frida Brochs:** Conceptualization, Methodology, Writing – review & editing. **Matilda Fahlen:** Conceptualization, Methodology, Writing – review & editing. **Kristoffer Strålin:** Conceptualization, Methodology, Writing – review & editing. **Therese Djärv:** Conceptualization, Methodology, Investigation, Writing – review & editing, Visualization, Supervision.

## Declaration of competing interest

The authors declare that they have no known competing financial interests or personal relationships that could have appeared to influence the work reported in this paper.
