# Supplementary material for: In-hospital cardiac arrest due to sepsis – Aetiologies and outcomes in a Swedish cohort study
Source: Resusc Plus. 2023 Nov 1;16:100492. doi: 10.1016/j.resplu.2023.100492 (PMC10641544; doi:10.1016/j.resplu.2023.100492)
Supplement: Supplementary Data 1 [file mmc1.docx]

**Supplementary table A. Overview of cultures for 123 patients with sepsis-related in-hospital cardiac arrest at Karolinska University Hospital 2007-2022.**

|  | **Blood culture**  **(n=107)** | | **Urine culture (n=90)** | | **Sputum culture (n=31)** | | **NPH culture (n=39)** | | **CVC culture (n=25)** | | **Intravenous access culture (n=23)** | | **Faecal culture (n=14)** | | **Joint fluid culture (n=10)** | |
| --- | --- | --- | --- | --- | --- | --- | --- | --- | --- | --- | --- | --- | --- | --- | --- | --- |
|  | **Alive** | **Dead** | **Alive** | **Dead** | **Alive** | **Dead** | **Alive** | **Dead** | **Alive** | **Dead** | **Alive** | **Dead** | **Alive** | **Dead** | **Alive** | **Dead** |
| **TOTAL Gram+ bacteria** | 2 | 22 | 1 | 8 | 1 | 7 | 2 | 7 | -- | 3 | 2 | 6 | -- | -- | 1 | 3 |
| -Staphylococcus aureus | 1 | 6 |  |  | 1 | 3 | 2 | 2 |  | 1 | 1 | 3 |  |  |  | 2 |
| -Strepotcoccus pneumonia | 1 | 6 |  |  |  |  |  | 2 |  |  |  | 1 |  |  |  | 1 |
| -Staphylococcus epididermidis |  | 6 |  |  |  |  |  |  |  | 2 |  | 1 |  |  |  |  |
| -Antigen of pneumococci |  |  |  | 3 |  |  |  |  |  |  |  |  |  |  |  |  |
| -Enterococcus faecalis |  | 4 | 1 | 1 |  | 1 |  |  |  |  | 1 |  |  |  |  |  |
| -Other Gram positive |  |  |  | 4 |  | 3 |  | 3 |  |  |  | 1 |  |  | 1 |  |
|  |  |  |  |  |  |  |  |  |  |  |  |  |  |  |  |  |
| **TOTAL Gram – bacteria** | 2 | 8 | 3 | 17 | 1 | -- | -- | 2 | -- | 2 | 2 | -- | -- | -- | -- |  |
| -Escherichia coli | 2 | 8 | 1 | 12 |  |  |  |  |  | 2 | 2 |  |  |  |  |  |
| -Klebsiella pneumonia |  |  | 2 | 3 |  |  |  |  |  |  |  |  |  |  |  |  |
| -Other G negative |  |  |  | 2 | 1 |  |  | 2 |  |  |  |  |  |  |  | 1 |
|  |  |  |  |  |  |  |  |  |  |  |  |  |  |  |  |  |
| **Others, <5 per agens*** | 4 |  | 1 | 9 | 2 | 2 |  |  |  | 3 |  | 1 | 1 | 2 |  | 1 |
| **Candida albicans and other fungi** |  |  |  |  | 1 | 5 |  |  |  | 1 |  |  |  |  |  |  |
| **Negative culture** | 10 | 46 | 10 | 38 | 4 | 2 | 3 | 26 | 4 | 11 | 2 | 5 | 1 | 8 |  | 1 |
| **Missing response** | 3 | 13 | 6 | 27 |  |  | 16 | 67 |  |  |  |  |  |  |  |  |

**Supplementary figure A. Sepsis-related IHCA at Karolinska University Hospital from 2007-2022.**

**Supplementary Table B. Cerebral Performance Category (CPC) at admission to hospital and at discharge from hospital among patients with sepsis-related IHCA surviving 30 days at Karolinska University Hospital from 2007-2022.**

|  | | | |  |  |
| --- | --- | --- | --- | --- | --- |
|  | **CPC at discharge** | | | |  |
| **CPC at admission** | 1 | 2 | 3 | 4 | Missing |
|  | | | | |  |
| 1 | 9 | 1 | 1 |  | 4 |
| 2 |  | 1 |  |  |  |
| 3 |  |  | 4 |  |  |
| 4 |  |  |  |  |  |
| Missing | 1 |  |  |  |  |

*NB! Green background corresponds to the same CPC at discharge compared to CPC at admission to hospital.*

*The red box captures those commonly categorised as good neurological outcome,*[*^22^*](#_ENREF_22) *i.e. CPC 1-2 at discharge.*
